# Comparative and synergistic impacts of lime and biochar on soil properties, nitrogen transformation, and microbial function in acidic soils under tobacco cropping

**DOI:** 10.3389/fpls.2025.1530128

**Published:** 2025-02-07

**Authors:** Bianhong Zhang, Lina Tang, Zhicheng Chen, Xiaoyan Chen, Lindong You, Ruixin Pan, Ting Chen, Yifei Liu, Wenxiong Lin, Jinwen Huang

**Affiliations:** ^1^ Key Laboratory for Genetics Breeding and Multiple Utilization of Crops, Ministry of Education/College of Agriculture, Key Laboratory of Crop Ecology and Molecular Physiology (Fujian Agriculture and Forestry University), Fujian Agriculture and Forestry University, Fuzhou, Fujian, China; ^2^ The Soil and Fertilization Research Laboratory, Tobacco Science Research Institute of Fujian Tobacco Monopoly Bureau, Fuzhou, Fujian, China; ^3^ College of JunCao Science and Ecology, Fujian Agriculture and Forestry University, Fuzhou, China

**Keywords:** lime, biochar, acidic soil, nitrogen utilization efficiency, tobacco

## Abstract

**Introduction:**

Lime and biochar are widely utilized to enhance nitrogen utilization in crops grown on acidic soils, though each has its own set of limitations. Understanding their combined effects is crucial for optimizing soil remediation strategies.

**Methods:**

This study investigates the impact of lime and biochar on nitrogen utilization efficiency (NUE) in a tobacco monoculture system, which has been practiced for 20 years on acidified soils in Fuzhou, southeastern China, over the period from 2021 to 2022. Four treatments were applied: control (CK), lime alone (L), biochar alone (B), and a lime-biochar combination (L+B).

**Results:**

The results indicated that all treatments significantly improved NUE, with increases ranging from 20.07% to 27.17% compared to CK. Biochar (B) was more effective than lime (L), and the combined treatment (L+B) showed comparable effects to biochar alone. Correlation analysis revealed that increases in soil pH and exchangeable base cations facilitated nitrogen transformation, thereby enhancing NUE. Lime treatments (L, L+B) promoted nitrification potential in rhizosphere soil, whereas biochar application (B, L+B) resulted in elevated nitrate nitrogen content. Microbial functional analysis indicated that lime (L, L+B) enhanced nitrification, while biochar (B, L+B) fostered dissimilatory nitrate reduction, thereby improving nitrogen retention. Pearson correlation analysis demonstrated a strong positive relationship between dissimilatory nitrate reduction and both soil alkali-hydrolyzable nitrogen and nitrate nitrogen contents.

**Conclusion:**

These findings suggest that lime enhances nitrification, while biochar promotes nitrate retention, together increasing soil nitrogen availability. The combined application of lime and biochar integrates these benefits, yielding results comparable to biochar alone. This study offers valuable insights into the synergistic use of lime and biochar for mitigating soil acidification and optimizing nitrogen management in agricultural systems.

## 1 Introduction

Soil acidification has emerged as a significant global issue in agricultural soil degradation, affecting approximately 40% of arable land (Wang et al., 2021). In China, the average soil pH has decreased by 0.5 units over the past three decades, with approximately 22.7% of arable land exhibiting a pH below 5.5. Projections indicate that crop production losses due to soil acidification will increase from 4% to 24% between 2010 and 2050 (Chu et al., 2020; Zhu et al., 2020). A primary contributor to this trend is the excessive application of nitrogen fertilizers (Zeng et al., 2017). Over-fertilization accelerates nitrification, producing H^+^ ions that replace alkaline base cations, thereby exacerbating soil acidification (Lu et al., 2014). Moreover, prolonged acidification results in elevated concentrations of H^+^ and Al^3+^, which severely impair root development, reduce nutrient absorption capacity, and ultimately lower nitrogen utilization efficiency (NUE), creating a detrimental feedback loop (Dos Reis et al., 2018; Yamamoto, 2019).

Beyond the reduction in crop nutrient absorption capacity, the depletion of available nitrogen in the soil is another key factor contributing to decreased NUE. Zhang et al. (2018) observed a significant positive correlation between soil pH and available nitrogen, which is largely attributed to the direct influence of pH changes on the activity of nitrogen-cycling microorganisms in the rhizosphere (Wang et al., 2017). Nitrogen availability in soil is primarily governed by ammonification and nitrification processes mediated by nitrogen-cycling microorganisms. The former process converts organic nitrogen into ammonium, while the latter transforms ammonium into nitrate nitrogen (Bernhard et al., 2005; Isobe et al., 2020). Studies have demonstrated that soil acidification, induced by excessive nitrogen application, significantly impairs nitrogen transformation efficiency, particularly when the pH drops below 5.5. At this point, the activity of nitrogen-cycling microorganisms in the rhizosphere is notably inhibited, thus limiting ammonification and nitrification (Sahrawat, 2008; Hao et al., 2020; Meng et al., 2023). Furthermore, He et al. (2024) emphasized that the reduced rates of nitrogen transformation in acidic soils result in insufficient nutrient supply for crops, leading to NUE significantly lower than those observed in alkaline soils, with a difference ranging from 1.1 to 2.1 times. Therefore, optimizing nitrogen cycling within the crop rhizosphere to enhance NUE has become a central focus of agricultural research, providing new directions for sustainable agricultural development.

Recent studies have increasingly focused on mitigating soil acidification and optimizing nitrogen cycling in agricultural systems. Lime, a conventional soil amendment, is widely used for acidification control due to its efficacy in providing alkaline ions such as calcium, magnesium, and potassium, which neutralize soil acidity and raise pH levels (Holland et al., 2019). Beyond its pH-raising effects, lime application has been shown to significantly enhance NUE in crops (Ai et al., 2015; Ylivainio et al., 2024) and to influence various aspects of nitrogen cycling in acidic soils. These include improved ammonification, nitrogen fixation, and nitrification, as well as a reduction in nitrous oxide (N_2_O) emissions (Feng et al., 2003; Fuentes et al., 2006; Kaushal et al., 2006). However, long-term lime applications can also result in negative outcomes, such as antagonistic effects on the absorption of other cations (e.g., Mg^2+^ and K^+^) (Alvarez et al., 2009) and risks of soil compaction and re-acidification (Du et al., 2020; Liu et al., 2023).

In contrast, biochar, an emerging soil amendment, has gained increasing attention due to its unique physical and chemical properties that can substantially improve soil quality and promote plant growth. Biochar’s porous structure enhances soil physical properties and nutrient retention (Wang et al., 2017), while also providing an ideal habitat for soil microorganisms. This, in turn, boosts microbial activity and diversity, creating a favorable microenvironment for the growth and proliferation of nitrogen-fixing, nitrifying, and denitrifying bacteria (Yan et al., 2019). These microbial processes play a critical role in enhancing nitrogen cycling and improving NUE in crops. Several studies have demonstrated that biochar application can significantly enhance NUE in crops grown on acidic soils (Liu et al., 2021; Zhao et al., 2024). It is evident that biochar offers more sustainable benefits compared to lime (Zhang S. W. et al., 2023). However, its high production and transportation costs present barriers to widespread adoption in agricultural production (Dai et al., 2017). Thus, while both lime and biochar offer distinct benefits, they also face challenges in modern agricultural practices. There is an urgent need to develop effective strategies for the application of soil amendments to mitigate soil acidity and enhance crop productivity in agricultural ecosystems. In recent years, numerous studies have proposed combining soil amendments to leverage their respective advantages and enhance land productivity. For example, combinations of lime and gypsum, or biochar with manure or straw, have demonstrated significant improvements in soil quality and productivity (Wu et al., 2021; Bossolani et al., 2023).

Tobacco, a major economic crop in China, thrives in soils with a pH range of 5.5 to 6.5 (Zhang et al., 2016). However, recent studies indicate that less than 40% of tobacco-growing areas in China have soils within this optimal pH range (Sun et al., 2020). In many regions of southern China, soil pH values are even lower than 5.5 (Zha et al., 2022). In Fujian, one of China’s three major tobacco-growing provinces, the average soil pH is only 5.19 (Xie et al., 2023), with soil acidification continuing to intensify. Preliminary research by our team has shown that the individual application of either lime or biochar significantly alleviates soil acidification and enhances tobacco productivity (Huang et al., 2023; Zhang et al., 2023). However, comprehensive research on the physiological mechanisms underlying the effects of lime and biochar applications in improving soil nitrogen cycling, mitigating soil acidification, and promoting soil-plant nitrogen transformations remains limited. Furthermore, studies on the synergistic effects of these amendments are scarce.

In light of these gaps, the present study hypothesizes that lime and biochar exert significantly different effects on improving acidic soils and enhancing NUE, and that their combined use may offer complementary benefits, more effectively improving the soil environment and promoting crop growth. The study focuses on soils acidified by long-term tobacco-rice cropping systems and examines the individual and combined effects of lime and biochar on the physicochemical properties of tobacco soils, nitrogen transformations in the soil-plant system, and the functional dynamics of rhizosphere nitrogen-cycling microbial communities. The objective is to provide scientific evidence to optimize soil improvement strategies, increase crop productivity in tobacco-growing regions, and promote the sustainable development of agricultural ecosystems.

## 2 Materials and methods

### 2.1 Experimental design

This study utilized the main tobacco variety “Yunyan 87” from Fujian Province, with the experimental materials provided by the Tobacco Research Institute of the Chinese Academy of Agricultural Sciences in Yunnan and the Tobacco Science Research Institute of the China National Tobacco Corporation in Fujian. The field experiment was conducted from 2021 to 2022 at the Agricultural Research Base of the Fujian Academy of Agricultural Sciences in Huangxi Town, Jin’an District, Fuzhou, Fujian Province (119°36′86″E, 26°17′33″N), over a two-year period. The experimental soil was red loam paddy soil that has been continuously used for tobacco-rice rotational cropping for 20 years. During the 2021 tobacco growing season, the effective accumulated temperature was 2445.59°C, with 459.11 mm of rainfall; in 2022, the effective accumulated temperature was 2209.04°C, with 610.43 mm of rainfall. The initial soil pH was 4.96, with organic matter content of 27.65 g·kg-1, total nitrogen content of 2.06 g·kg^-1^, total phosphorus content of 0.88 g·kg^-1^, total potassium content of 24.91 g·kg^-1^, alkali-hydrolyzable nitrogen content of 96.37 mg·kg^-1^, available phosphorus content of 51.10 mg·kg^-1^, and available potassium content of 190.06 mg·kg^-1^.

From January to August 2021, a randomized block design was applied, with four treatments: a control (CK) with no soil amendments, 1500 kg·ha^-1^ lime (L), 30 t·ha^-1^ biochar (B), and a combination of 750 kg·ha^-1^ lime + 15 t·ha^-1^ biochar (L+B). Each treatment was replicated three times, with an additional blank treatment (N0) with no amendments and nitrogen fertilizer, used for calculating NUE. The application rates of lime and biochar were based on the results of our previous studies (Huang et al., 2023; Zhang et al., 2023). Each experimental plot had an area of 144 m^2^ (24 m long, 6 m wide), with a total of 12 plots. One month before planting, amendments were evenly applied to the soil surface, followed by rotary tilling and ridge formation. The ridges were 35 cm high, with row spacing of 1.2 m × 0.5 m. Fertilization rates were 127.5 kg·ha^-1^ N, 99 kg·ha^-1^ P_2_O_5_, and 402 kg·ha^-1^ K_2_O. Except for the different treatments, all other field management practices followed the high-yield, high-quality cultivation measures of Fujian Province. In 2022, the experiment was repeated, with additional analyses on nitrogen metabolism enzyme activity in plants, root vitality, soil physicochemical properties, available nitrogen content in soil, urease activity and nitrification potential in rhizosphere soil, and the diversity and nitrogen-cycling function of rhizosphere bacterial communities.

The biochar used in this study was derived from tobacco stalks produced by the Henan Shangqiu Sanli New Energy Co., Ltd., which was subjected to high-temperature pyrolysis (~450°C) in an oxygen-limited environment. Its physicochemical properties were as follows: pH 9.66, fixed carbon content 475.90 g·kg^-1^, total nitrogen 15.00 g·kg^-1^, total phosphorus 1.40 g·kg^-1^, and total potassium 20.10 g·kg^-1^. The lime used had primary components of calcium oxide and calcium carbonate, with a pH of 11.41, total phosphorus content of 0.07 g·kg^-1^, and total potassium content of 0.48 g·kg^-1^.

### 2.2 Measurement items and methods

#### 2.2.1 Determination of nitrogen utilization efficiency in tobacco plants

At the harvest stage, three representative plants exhibiting uniform growth were selected from each plot. These plants were then separated into roots, stems, and leaves, which were subjected to a deactivation process at 105°C for 30 minutes. Following this, the samples were dried at 80°C until a constant weight was achieved, allowing for the determination of dry matter weight. After grinding the dried samples through a 0.25 mm sieve, total nitrogen was extracted from the plant tissues using a concentrated sulfuric acid-hydrogen peroxide digestion method. Nitrogen content was subsequently analyzed using the Smartchem 2000 automated chemical analyzer (Germany). Nitrogen accumulation and NUE were calculated using the following formulas:


$$\begin{array}{c} \text{Nitrogen accumulation per unit area of plant }\left({\text{kg}\cdot \text{ha}}^{-1}\right)\\ =\text{Nitrogen accumulation per plant}\times \text{planting density} \end{array}$$

$$\begin{array}{c} \text{Nitrogen utilization efficiency}\;\text{(NUE)}\;\left(\%\right)\\ =[(\text{Nitrogen uptake by plants in nitrogen treatment area}\\ -\text{Nitrogen uptake by plants in the area without}\;\\ \text{nitrogen treatment})/\text{Nitrogen application rate}]\times 100\% \end{array}$$

$$\begin{array}{c} \text{Nitrogen harvest index}\left(\text{NHI}\right)\left(\%\right)\\ =\text{(Nitrogen accumulation in plant leaves}/\\ \text{Whole plant Nitrogen accumulation)}\times 100\% \end{array}$$

#### 2.2.2 Determination of nitrogen metabolism enzymes in tobacco leaves and root vitality

During the root elongation, vigorous growth, and topping stages, three representative tobacco plants exhibiting uniform growth were selected from each plot. Sampling was carried out on sunny days between 9:00 and 10:30 AM. Samples of the upper functional leaves and roots from each treatment were collected, with the main veins of the leaves removed. Fresh samples were immediately stored in liquid nitrogen and transported to the laboratory for further analysis. Nitrate reductase (NR) activity was measured using a nitrate reductase assay kit from Solarbio (Bories and Bories, 1995). Glutamine synthase (GS) activity was determined using a glutamine synthase assay kit from Solarbio (Bressler and Ahmed, 1984). Root vitality (RV) was assessed using the triphenyl tetrazolium chloride (TTC) method (Li et al., 2021).

#### 2.2.3 Determination of urease activity and nitrification potential in rhizosphere soil

Soil sampling for analysis was carried out during the root elongation, vigorous growth, and topping stages. Soil samples were collected from near the roots (within 5 mm) using a root shaking method, with three replicates taken from each plot. The samples were stored in a cool, dry environment until air-dried, after which they were ground and sieved through a 100-mesh screen for subsequent analysis. Urease (URE) activity in the rhizosphere soil was measured using the phenol-sodium hypochlorite colorimetric method (Li et al., 2016), while nitrification potential (NP) was assessed using the suspension culture method (Norton and Stark, 2011).

#### 2.2.4 Determination of the physicochemical properties of bulk soil

During the vigorous growth stage of tobacco, one sampling point was established on the ridge surface between two consecutive representative tobacco plants in each plot. Soil bulk density (BD), total porosity (POR), and field moisture capacity (FC) were measured at soil depths of 0-10 cm, 10-20 cm, and 20-30 cm using cutting ring method. The average values of these parameters were then calculated for the 0-30 cm soil layer. Each treatment was replicated three times (Zhang et al., 2023). Soil samples were collected from each plot using the five-point sampling method, with five soil cores taken per plot. Prior to mixing, the soil cores from the same depth were combined into a single composite sample for each replicate. Three replicates were collected from each treatment. Soil samples from the 0-30 cm depth were used to determine soil chemical properties and soil nutrients. The samples were air-dried indoors, ground, and sieved through a 1 mm mesh. Soil pH was determined using a potentiometric method, while oxidation-reduction potential (ORP) was measured with a WP-T100 oxidation-reduction potential meter. Exchangeable acidity (EA) was quantified using the potassium chloride extraction method followed by neutralization titration, and exchangeable base cation (EB) concentrations were measured using the ammonium acetate extraction method with neutralization titration. The sum of exchangeable acidity and base cations was used to calculate the soil cation exchange capacity (CEC) (Lu, 2000).

#### 2.2.5 Determination of available nitrogen content in bulk soil

Soil samples were collected at four growth stages—root elongation, vigorous growth, topping, and harvesting—using the five-point sampling method outlined in Section 2.2.4. The content of alkali-hydrolyzable nitrogen (AN) was quantified via the alkali diffusion method. Ammonium nitrogen (NH_4_^+^-N) was measured using the indophenol blue colorimetric technique, while nitrate nitrogen (NO_3_^–^N) was determined using the dual-wavelength spectrophotometric method (Lu, 2000). Microbial biomass nitrogen (MBN) was estimated using the chloroform fumigation-extraction method, with a conversion factor of 0.45 (Brookes et al., 1985).

#### 2.2.6 Rhizosphere soil bacterial DNA extraction and high-throughput sequencing

During the vigorous growth stage, three representative plants exhibiting uniform growth were selected from each experimental treatment. The entire root system was excavated, and debris surrounding the rhizosphere was rapidly removed. The soil adhering to the roots was wrapped in aluminum foil, mixed, and immediately frozen in liquid nitrogen before being transferred to a -80°C freezer for storage. The extraction of soil DNA, PCR amplification, and sequencing were performed by Allwegene Technology Co., Ltd (Beijing, China). Soil samples were weighed, and total microbial DNA was extracted. The concentration and quality of the DNA were assessed using a NanoDrop 2000 spectrophotometer and agarose gel electrophoresis, after which the DNA samples were stored at -20°C. Following DNA extraction, the 16S rDNA V3-V4 region was amplified using barcoded primers 338F (5’ -ACTCCTACGGGAGGCAGCAG-3’) and 806R (5’-GGACTACHVGGGTWTCTAAT-3’). Library construction was carried out using the TruSeq^®^ DNA PCR-Free Sample Preparation Kit, and sequencing was performed on the Illumina MiSeq platform. The raw image data files obtained from sequencing were processed using the RDP Classifier algorithm and the Silva database (Release 119) to cluster reads with greater than 97.0% similarity, generating representative sequences for operational taxonomic units (OTUs). The community composition was annotated, and the abundance of OTUs across samples was normalized for subsequent diversity and differential analysis (NCBI accession number: PRJNA1118945).

### 2.3 Statistical analysis

Alpha species diversity of the community was calculated using the diversity function from the R package “vegan” (Dixon, 2003). Beta diversity was assessed by computing the Weighted-Unifrac distances between samples, followed by Principal Coordinates Analysis (PCoA). Species annotation of feature sequences was performed using the PICRUSt2 tool, and potential functional genes within the samples were identified via the Kyoto Encyclopedia of Genes and Genomes (KEGG) database (Douglas et al., 2020). Furthermore, nitrogen cycling-related functional gene information was extracted using the DiTing software (Gao et al., 2024). The abundance of these functional genes was subsequently normalized using Z-score transformation. Figures and tables were generated using Microsoft Excel 2019, Origin 2024b, and R 4.2.9. Statistical analyses were conducted with SPSS 22.0, utilizing Duncan’s multiple range tests at α = 0.05 to assess significance.

## 3 Results

### 3.1 Effects of different treatments on NUE and outputs of tobacco plants

As shown in **Table 1**, the nitrogen accumulation and NUE of tobacco plants were significantly higher in all experimental treatments compared to CK treatment after the application of soil amendments. Over the two-year period, NUE was significantly increased by 20.31% to 29.10% in year one and by 19.84% to 25.24% in year two, compared to the CK treatment. In addition, NHI was also significantly higher in all treatments relative to CK. Among the treatments, the B treatment exhibited significantly higher NUE than the L treatment, with increases in nitrogen accumulation of 5.00% and 7.54% in the two years, respectively, and NUE improvements of 8.79% and 5.40%. The L+B treatment showed no significant difference in NUE compared to the B treatment. Regarding yield value, both the B treatment and the L+B treatment demonstrated superior performance compared to the L treatment (**Supplementary Figure S1**). In conclusion, the results over the two years clearly indicate that all treatments significantly enhance tobacco yield value and NUE, although the extent of improvement varies. Notably, the L+B treatment achieved significantly greater enhancement compared to the L treatment, with no significant difference when compared to the B treatment. This finding underscores the importance of adopting a comprehensive approach to soil improvement strategies.

**Table 1:** Nitrogen accumulation and nitrogen utilization efficiency of tobacco plants under different treatments.

Year	Treatment	Leaf (kg·ha^-1^)	Stem (kg·ha^-1^)	Root (kg·ha^-1^)	Whole plant (kg·ha^-1^)	NUE (%)	NHI (%)
2021	N0	20.98 ± 1.79d	9.76 ± 0.86c	9.12 ± 1.34c	39.86 ± 2.38d		
	CK	34.56 ± 2.15c	18.46 ± 1.44b	21.04 ± 2.43b	74.07 ± 5.39c	26.83 ± 4.28c	46.66 ± 0.85b
	L	51.96 ± 2.23b	25.08 ± 1.09a	28.82 ± 2.90a	105.86 ± 2.10b	47.14 ± 3.11b	49.08 ± 1.49a
	B	55.80 ± 2.07a	25.60 ± 1.53a	29.76 ± 2.03a	111.16 ± 1.61a	55.93 ± 1.09a	50.20 ± 0.85a
	L+B	54.22 ± 1.28a	24.13 ± 1.91a	29.11 ± 1.97a	107.46 ± 1.99ab	53.02 ± 3.32a	50.46 ± 1.87a
2022	N0	19.21 ± 1.22d	5.99 ± 0.22c	6.04 ± 0.45b	31.23 ± 1.03d		
	CK	28.33 ± 1.24c	16.33 ± 1.07b	21.38 ± 1.91a	66.04 ± 1.73c	27.30 ± 1.36c	42.90 ± 1.23b
	L	47.54 ± 0.43b	22.24 ± 0.53a	21.55 ± 2.17a	91.33 ± 1.51b	47.14 ± 1.18b	52.05 ± 1.09a
	B	53.73 ± 1.24a	20.62 ± 0.56a	23.87 ± 1.47a	98.22 ± 0.60a	52.54 ± 0.47a	54.70 ± 1.59a
	L+B	51.16 ± 1.05a	20.63 ± 1.20a	22.99 ± 0.56a	94.78 ± 0.41ab	49.84 ± 1.05a	53.98 ± 2.58a

Nitrogen utilization efficiency for the CK, L, B, and L+B treatments were calculated using N0 as the control. NUE, Nitrogen utilization efficiency; NHI, Nitrogen harvest index; N0, control without nitrogen fertilization; CK, control without soil amendment; L, lime-alone treatment; B, biochar-alone treatment; L+B, lime and biochar combined treatment. Data depicts means ± SD of three biological replicates. Significant differences between treatments (*P<* 0.05) are illustrated by different lowercase letters.

### 3.2 Effects of different treatments on the physicochemical properties in the bulk soil of tobacco

As shown in **Table 2**, all experimental treatments significantly increased soil pH, reduced EA, and significantly enhanced EB and CEC compared to the CK treatment. Among the treatments, the B and L+B treatments were significantly more effective than the L treatment in increasing pH and EB. Additionally, both the B and L+B treatments also significantly improved ORP. Regarding soil physical properties, as illustrated in **Supplementary Figure S2**, both the B and L+B treatments significantly reduced BD and increased FC compared to the CK treatment. In contrast, the L treatment showed no significant difference in BD and FC when compared to CK. These findings suggest that the application of soil amendments can effectively alleviate soil acidification, shifting the soil environment towards a more alkaline condition. Furthermore, compared to the L treatment, both the B and L+B treatments demonstrated more pronounced effects in enhancing soil pH and improving soil aeration.

**Table 2:** Chemical properties of tobacco soil in under different each soil treatment.

	pH	EA (mmol·kg^-1^)	EB (mmol·kg^-1^)	CEC (mmol·kg^-1^)	ORP (mV)
CK	4.89 ± 0.01d	12.83 ± 0.17a	29.84 ± 0.04c	42.67 ± 0.15c	639.48 ± 13.33c
L	5.04 ± 0.02c	8.33 ± 0.01b	35.50 ± 0.07b	43.84 ± 0.11b	641.67 ± 12.97c
B	5.27 ± 0.01a	7.00 ± 0.17c	37.12 ± 0.11a	44.32 ± 0.11a	728.23 ± 5.10a
L+B	5.13 ± 0.02b	7.17 ± 0.17c	37.34 ± 0.04a	44.50 ± 0.19a	689.04 ± 7.73b

EA, Exchangeable acidity; EB, Exchangeable base cations; CEC, Cation exchange capacity; ORP, Oxidation-reduction potential; CK, control without soil amendment; L, lime-alone treatment; B: biochar-alone treatment; L+B, lime and biochar combined treatment. Data depicts means ± SD of three biological replicates. Significant differences between treatments (*P<* 0.05) are illustrated by different lowercase letters.

### 3.3 Changes in plant-soil nitrogen transformation capacity

#### 3.3.1 Changes in nitrogen metabolism enzyme activity and RV in tobacco plants

**Figure 1:**
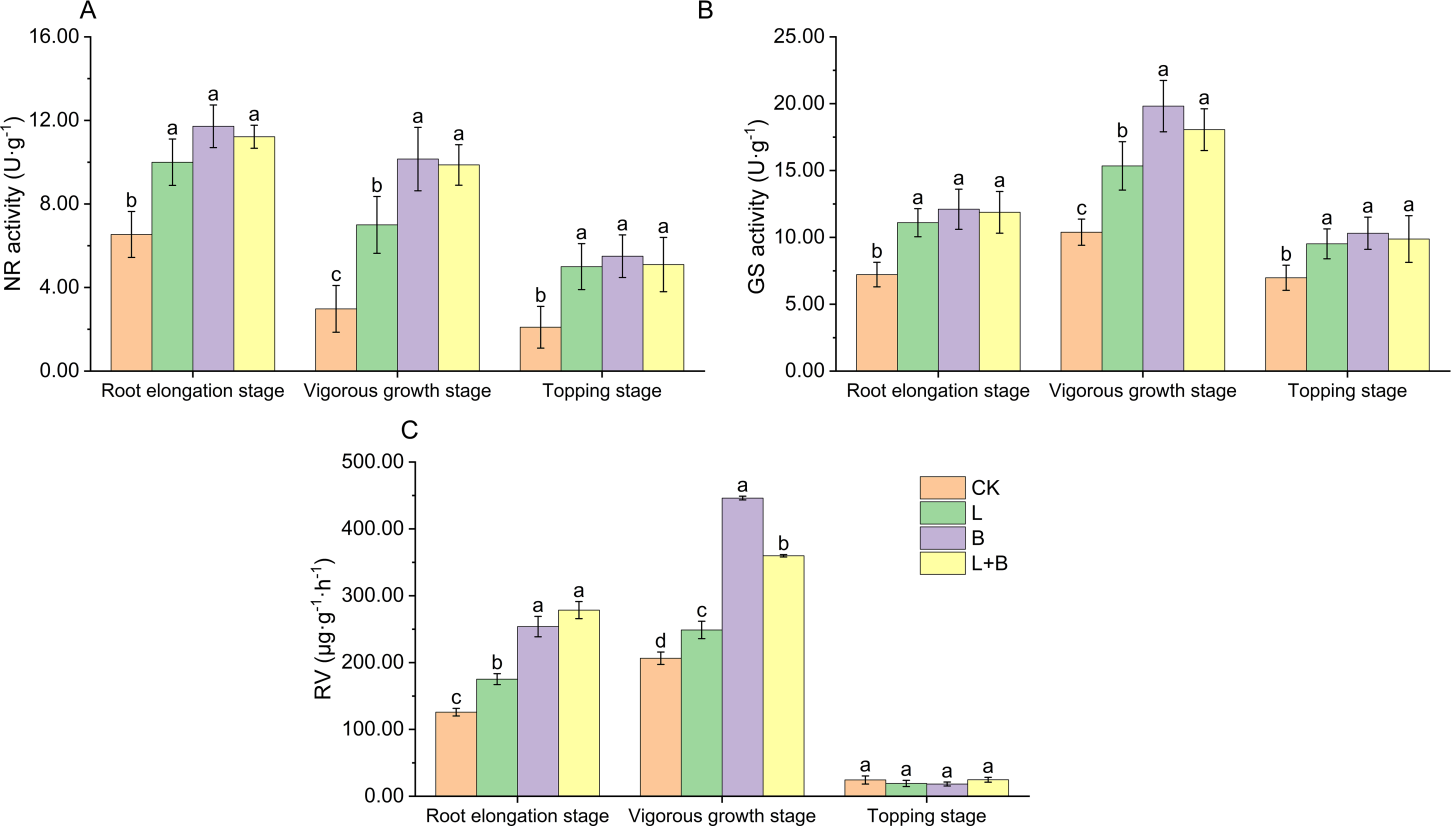
Nitrate reductase activity **(A)**, glutamine synthetase activity **(B)** of tobacco leaves, and root vitality **(C)** in each treatment. NR, Nitrate reductase; GS, Glutamine synthetase; RV, Root vitality; CK, control without soil amendment; L, lime-alone treatment; B, biochar-alone treatment; L+B, lime and biochar combined treatment. Data depicts means ± SD of three biological replicates. Significant differences between treatments (*P<* 0.05) are illustrated by different lowercase letters.

As shown in **Figure 1**, all experimental treatments significantly enhanced NR activity of tobacco leaves during the root elongation, vigorous growth, and topping stages compared to the CK treatment, with increases ranging from 52.90% to 79.20%, 134.90% to 240.60%, and 138.09% to 161.90%, respectively (**Figure 1A**). Additionally, the activity of GS was significantly elevated across all three stages, with increases of 53.74% to 67.73%, 47.73% to 90.76%, and 36.39% to 47.71%, respectively (**Figure 1B**). The increase in RV during the root elongation and vigorous growth stages ranged from 39.18% to 121.24% and 20.46% to 115.98%, respectively (**Figure 1C**). Notably, during the vigorous growth stage, the B and L+B treatments had a significantly greater effect on NR activity, GS activity, and RV compared to the L treatment. These results suggest that all experimental treatments significantly improve nitrogen assimilation efficiency and RV in tobacco plants, with the B and L+B treatments having particularly pronounced effects during the vigorous growth stage.

#### 3.3.2 Effects of different treatments on URE activity and NP in the rhizosphere soil of tobacco

**Figure 2:**
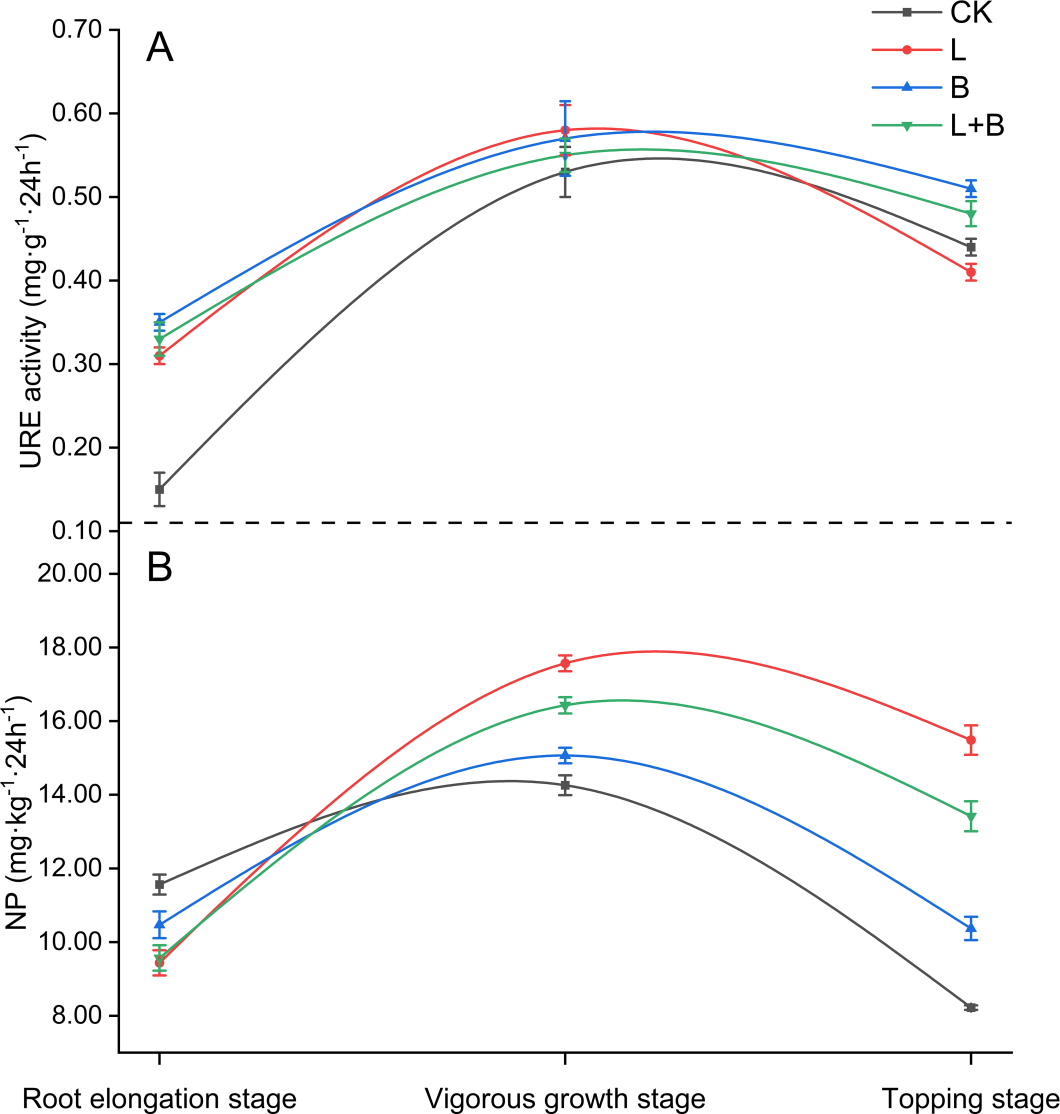
Urease activity **(A)** in tobacco rhizosphere soil and its nitrification potential **(B)**. URE, Urease; NP, Nitrification potential; CK, control without soil amendment; L, lime-alone treatment; B, biochar-alone treatment; L+B, lime and biochar combined treatment. Data depicts means ± SD of three biological replicates. Significant differences between treatments (*P<* 0.05) are illustrated by different lowercase letters.

URE activity and NP in soil are key indicators of the available nitrogen production capacity. As shown in **Figure 2**, during the root elongation stage of tobacco, URE activity in the rhizosphere under all experimental treatments was significantly higher than that in the CK treatment (**Figure 2A**). During the vigorous growth stage, URE activity in the tobacco rhizosphere reached its peak; however, no significant differences were observed among the various treatments (**Figure 2A**). The NP in the rhizosphere initially increased, then declined, with the highest levels occurring during the vigorous growth stage. Specifically, during the root elongation stage, NP in the rhizosphere of all experimental treatments was significantly lower than in the CK treatment. Conversely, during the vigorous growth stage, NP in all experimental treatments was significantly higher than in the CK treatment, with the L treatment and L+B treatment showing increases of 16.61% and 9.05%, respectively, compared to the B treatment (**Figure 2B**). These findings suggest that the application of soil amendments can effectively enhance the biochemical processes involved in nitrogen cycling within the soil. Notably, the L treatment had a significantly greater impact on nitrification potential than both the B and L+B treatments, with the L+B treatment exhibiting a secondary effect.

#### 3.3.3 Effects of different treatments on the variations in available nitrogen content in the bulk soil of tobacco

**Figure 3:**
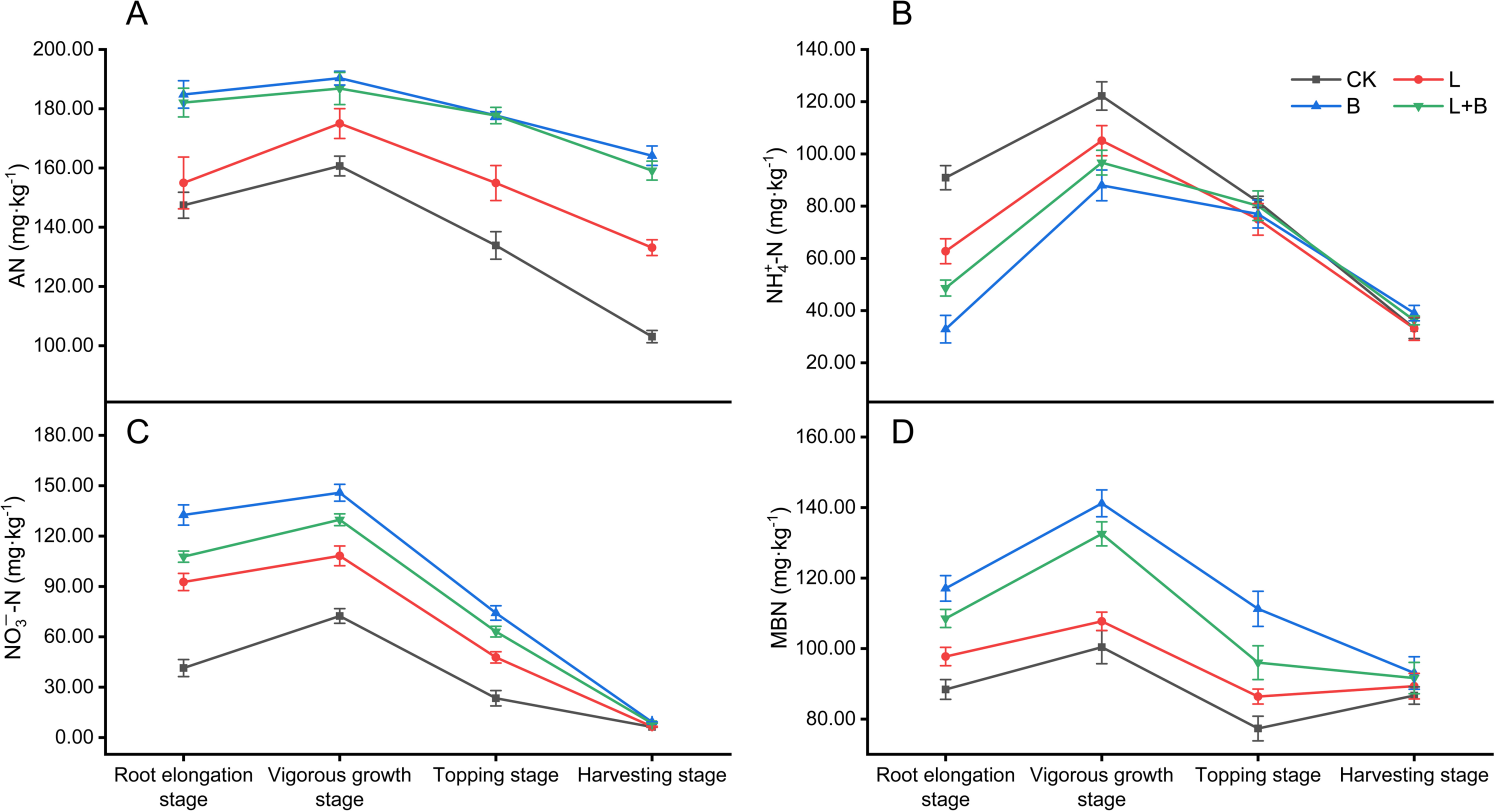
Contents of alkali-hydrolyzable nitrogen **(A)**, ammonium nitrogen **(B)**, nitrate nitrogen **(C)**, and microbial biomass nitrogen **(D)** in tobacco bulk soil. AN, Alkali-hydrolyzable nitrogen; NH_4_^+^-N, Ammonium nitrogen; NO_3_^−^-N, Nitrate nitrogen; MBN, Microbial biomass nitrogen; CK, control without soil amendment; L, lime-alone treatment; B, biochar-alone treatment; L+B, lime and biochar combined treatment. Data depicts means ± SD of three biological replicates. Significant differences between treatments (*P<* 0.05) are illustrated by different lowercase letters.

As illustrated in **Figure 3**, the content of AN in the soil reached its peak during the vigorous growth stage of tobacco. All experimental treatments resulted in a significant increase in AN content, ranging from 8.91% to 18.46%, as well as an increase in NO_3_^–^N content, which rose by 49.48% to 101.41%, compared to the CK treatment (**Figures 3A, C**). Additionally, these treatments significantly reduced NH_4_^+^-N content during the vigorous growth stage, with reductions ranging from 14.00% to 28.02% relative to the CK treatment (**Figure 3B**). Notably, both the B and L+B treatments significantly enhanced NO_3_^–^N content by 6.76% to 8.76% compared to the L treatment (**Figure 3C**). Furthermore, the B and L+B treatments also significantly increased both AN and MBN content relative to the L treatment (**Figures 3A, D**). These findings highlight the considerable impact of soil amendments on nitrogen cycling. Specifically, the L treatment was less effective in enhancing soil available nitrogen content compared to the B and L+B treatments.

#### 3.3.4 Correlation analysis of nitrogen utilization efficiency, plant-soil nitrogen transformation capacity, and soil physicochemical properties

**Figure 4:**
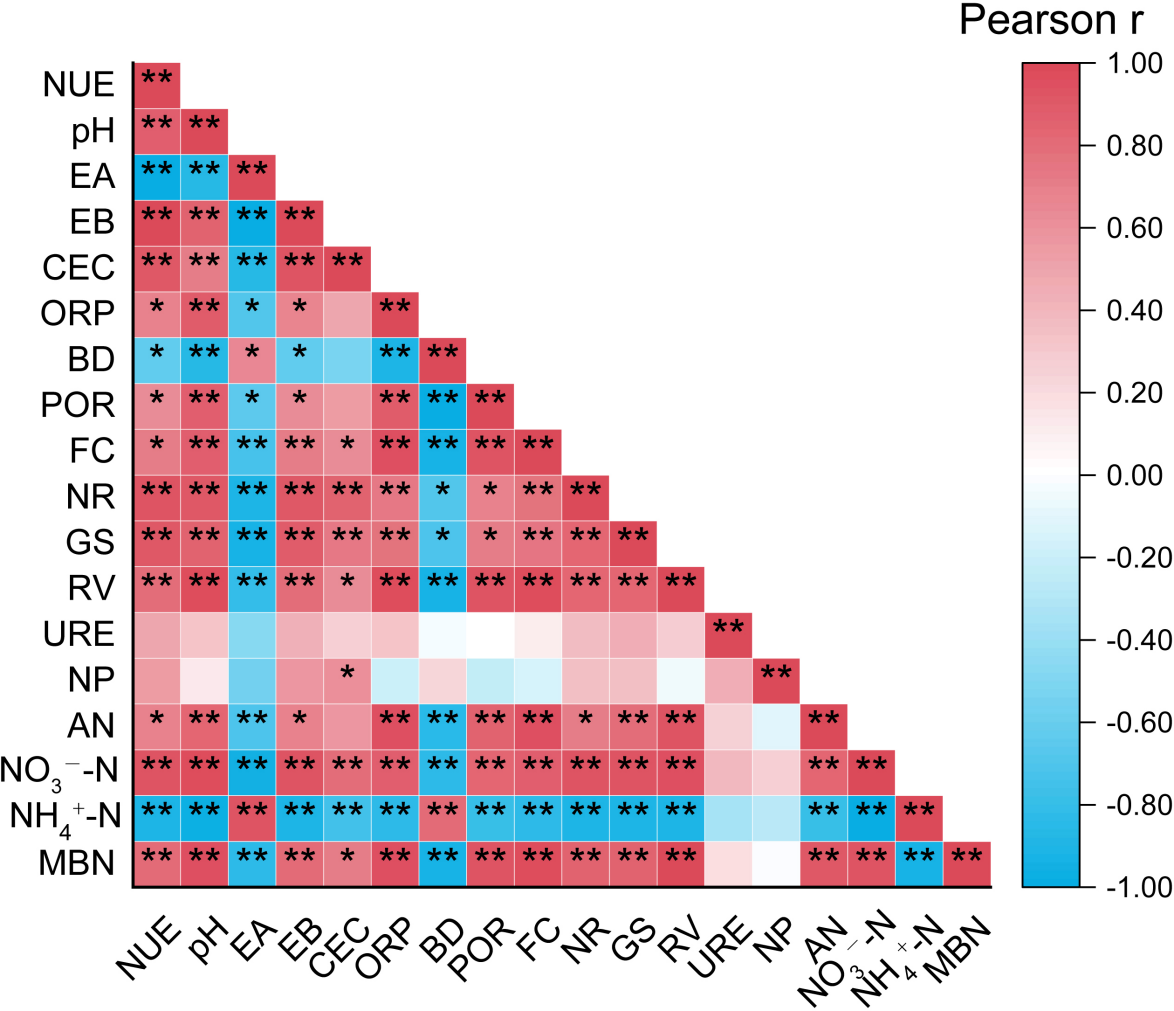
Pearson correlation analysis of nitrogen utilization efficiency in tobacco plants, plant-soil nitrogen transformation capacity, and soil physicochemical properties. “*” at 0.05 level (two-tailed), the correlation was significant; “**” at level 0.01 (two-tailed), the correlation was significant. NUE, Nitrogen utilization efficiency; EA, Exchangeable acidity; EB, Exchangeable base cations; CEC, Cation exchange capacity; ORP, Oxidation-reduction potential; BD, Bulk density; POR, Porosity; FC, Field moisture capacity; AN, Alkali-hydrolyzable nitrogen; NH_4_^+^-N, Ammonium nitrogen; NO_3_^−^-N, Nitrate nitrogen; MBN, Microbial biomass nitrogen.

A correlation analysis was performed to examine the relationships among NUE, soil physicochemical properties, and plant-soil nitrogen transformation capacity during the vigorous growth stage, with the results presented in **Figure 4**. NUE, NR, GS, and RV all showed a highly significant positive correlation with pH and EB (*P*< 0.01). Additionally, pH exhibited a highly significant positive correlation with AN, NO_3_--N, and MBN. Moreover, RV demonstrated a highly significant negative correlation with BD (*P*< 0.01). Notably, no significant correlation was observed between NP and NUE, nor between NP and NO_3_--N.

### 3.4 Evolution of nitrogen-cycling microbial communities in the rhizosphere of tobacco under different treatments

#### 3.4.1 Rhizosphere bacterial community diversity and species composition

As shown in **Supplementary Table S1**, the experimental treatments had varying effects on bacterial community species diversity. The L and L+B treatments significantly increased species diversity (observed OTUs, Ace, Chao1, and Shannon indices) compared to the CK treatment, while the B treatment did not show significant differences in species diversity relative to the CK treatment. As illustrated in **Supplementary Figure S3**, dimensionality reduction analysis revealed substantial differences in species composition between the experimental treatments and the CK treatment, with the differences between the L+B and L treatments, as well as between the L and B treatments, being more pronounced. This suggests that lime and biochar exert distinct effects on species composition, and their combined application may interact synergistically to enhance these effects.

Regarding species composition, as shown in **Supplementary Figure S4**, the L and L+B treatments significantly reduced the relative abundances of Proteobacteria and Bacteroidota, while significantly increasing the relative abundances of Chloroflexi and Acidobacteriota. In contrast, the B treatment significantly increased the relative abundances of Proteobacteria and Bacteroidota, and significantly decreased the relative abundances of Chloroflexi and Acidobacteriota (**Supplementary Figure S4A**). Furthermore, under the L treatment, the relative abundance of Nitrospirota was the highest among all treatments, followed by the L+B treatment. In terms of dominant genera, all experimental treatments significantly reduced the relative abundances of *Rhodanobacter* and *Chujaibacter* compared to the CK treatment (**Supplementary Figure S4B**).

#### 3.4.2 Analysis of the abundance of rhizosphere nitrogen-cycling functional genes and pathways

**Figure 5:**
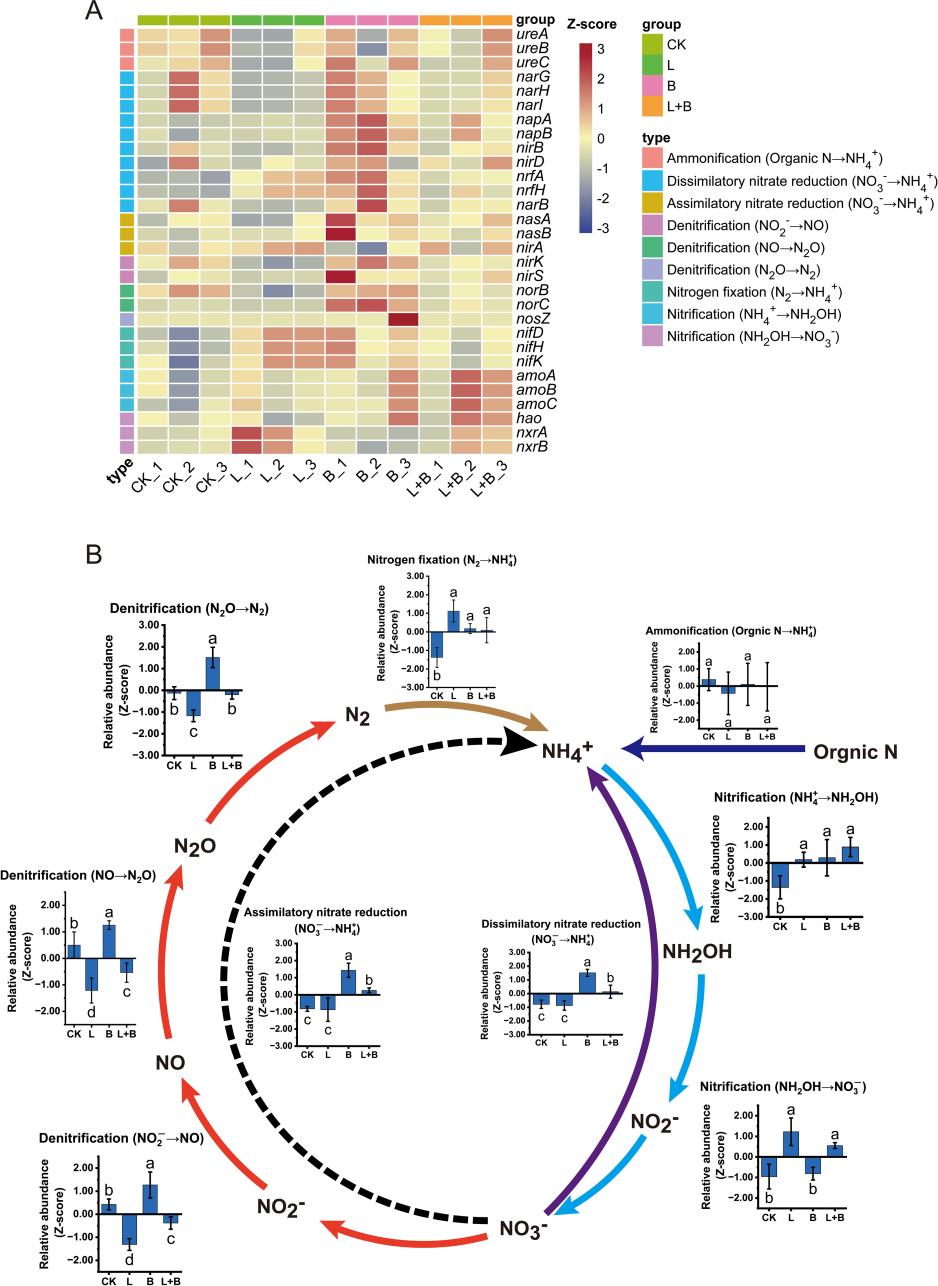
Relative abundance of nitrogen cycling functional genes **(A)** and associated nitrogen cycling pathways **(B)** in the tobacco rhizosphere under different treatments. Data were normalized using Z-score transformation. CK, control without soil amendment; L, lime-alone treatment; B, biochar-alone treatment; L+B, lime and biochar combined treatment. Data depicts means ± SD of three biological replicates. Significant differences between treatments (*P<* 0.05) are illustrated by different lowercase letters.

Although the changes in species composition mentioned above indicate that the experimental treatments significantly affected the microbial community structure, no clear alterations in nitrogen cycling functions were observed in these results. To further investigate this issue, we annotated the nitrogen cycling functional genes in the rhizosphere bacterial communities using the KEGG database, with the results presented in **Figure 5**. A total of 30 functional genes were identified, which were classified into six major categories based on their functional characteristics, encompassing nine distinct biological processes (**Figure 5A**). Further statistical analysis of the relative abundance of each nitrogen cycling pathway is shown in **Figure 5B**. The results demonstrate that different soil amendment treatments exhibited both common and divergent effects on soil nitrogen cycling processes. All experimental treatments significantly increased the functional abundance of nitrogen fixation and the first stage of nitrification (NH_4_^+^ → NH_2_OH) compared to the CK treatment. In the subsequent stage of nitrification (NH_2_OH → NO_3_-), the L and L+B treatments significantly outperformed the B and CK treatments. In the three stages of denitrification, the functional abundance followed the trend: B > CK > L+B > L, with the B treatment significantly higher than all other treatments, while the L and L+B treatments showed significantly lower functional abundances than the CK treatment, and the L treatment was significantly lower than the L+B treatment. Furthermore, the B treatment significantly enhanced the functional abundances of dissimilatory nitrate reduction and assimilatory nitrate reduction, while the L+B treatment also demonstrated significant increases in these two processes compared to the L and CK treatments. In contrast, the L treatment showed no significant difference from the CK treatment in these two processes.

In summary, all soil amendment treatments significantly increased the functional abundances of nitrogen fixation and initial nitrification in soil nitrogen cycling. The L and L+B treatments performed better than the B treatment in the subsequent stages of nitrification, while the B treatment showed the highest performance in denitrification and nitrate reduction processes, followed by the L+B treatment.

#### 3.4.3 Correlation between the functional abundance of rhizosphere nitrogen-cycling pathways and soil available nitrogen content and nitrogen utilization efficiency

**Figure 6:**
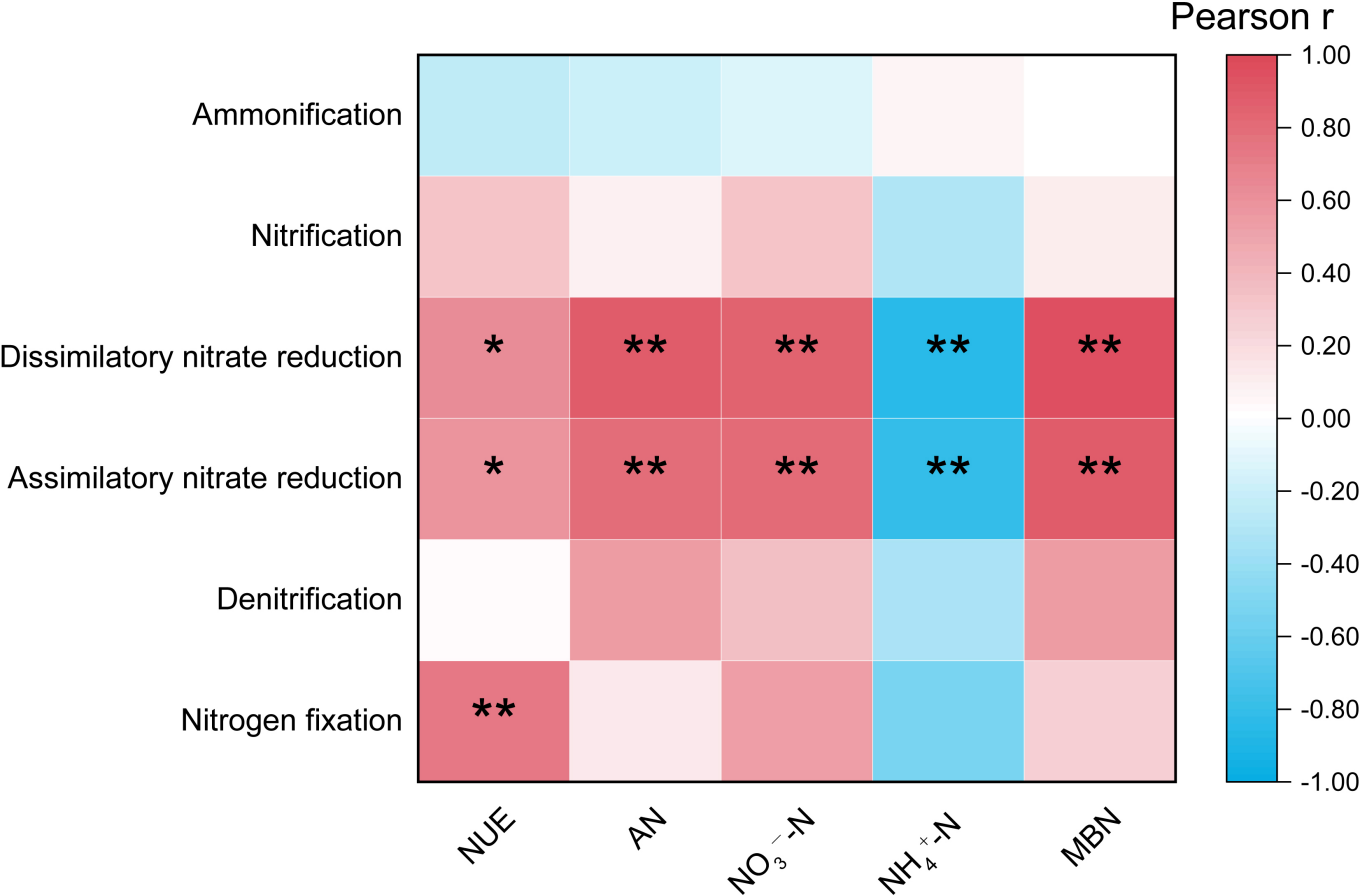
Pearson correlation analysis between nitrogen cycling functional abundance and soil nitrogen content regarding the nitrogen utilization efficiency ”*” at 0.05 level (two-tailed), the correlation was significant; “**” at level 0.01 (two-tailed), the correlation was significant. NUE, Nitrogen utilization efficiency; AN, Alkali-hydrolyzable nitrogen; NH_4_^+^-N, Ammonium nitrogen; NO_3_^−^-N, Nitrate nitrogen; MBN, Microbial biomass nitrogen.

As shown in **Figure 6**, a highly significant positive correlation was observed between nitrogen fixation and NUE (*P*< 0.01). Additionally, both dissimilatory nitrate reduction and assimilatory nitrate reduction exhibited a significant positive correlation with NUE (*P*< 0.05). Furthermore, these two nitrate reduction processes demonstrated highly significant positive correlations with the content of AN, NO_3_--N, and MBN (*P*< 0.01). In contrast, they showed a highly significant negative correlation with NH_4_^+^-N (*P*< 0.01).

## 4 Discussion

This study, conducted over a 2-year fixed-point trial, demonstrates that the application of lime (L), biochar (B), and their combination (L+B) significantly enhances nitrogen utilization efficiency and nitrogen harvest index in tobacco. Notably, the L+B treatment resulted in a more substantial improvement than the L treatment, with no significant difference observed when compared to the B treatment (**Table 1**). These findings align with those of Mosharrof et al. (2021) and Elbagory et al. (2024), who reported that the combined application of lime and biochar outperforms the individual application of either amendment in enhancing the productivity of maize and wheat. This suggests that the joint application of lime and biochar holds significant potential for improving nitrogen utilization in crops grown in acidic soils.

To further elucidate the mechanistic differences between lime and biochar in ameliorating acidic soils and enhancing nitrogen utilization efficiency in tobacco, this study analyzed soil physicochemical properties and nitrogen transformation capacities between plants and soil. The results revealed that all amendment treatments increased soil pH and the concentration of exchangeable base cations. However, compared to the L treatment, both the B and L+B treatments showed significantly greater effects on improving soil acidity, enhancing soil aeration, and increasing the oxidation-reduction potential (**Table 2**). This difference may be attributed to the slower mobility of alkaline ions in lime, which limits their rapid penetration into deeper soil layers (Conyers et al., 2003). Previous studies have suggested that lime typically requires a longer period to improve the acidity of deeper soil layers (Tang et al., 2013; Wang et al., 2016). In contrast, when lime and biochar are applied together, biochar facilitates the faster diffusion of alkaline ions from lime, thereby accelerating the amelioration of soil acidity. Consequently, the combined application of lime and biochar represents a promising strategy for effectively improving acidic soils.

Root vitality is a critical indicator of a plant’s nutrient absorption capacity (Oshunsanya et al., 2019), while leaf nitrate reductase and glutamine synthetase are key enzymes influencing nitrogen assimilation and uptake (Anas et al., 2020). Our results indicate that, compared to the L treatment, both the B and L+B treatments significantly enhanced root vitality, as well as the activities of leaf nitrate reductase and glutamine synthetase (**Figure 1**). We suggest that biochar application reduces soil resistance, improves soil permeability, and increases water retention capacity, thereby promoting tobacco root growth (Asai et al., 2009). This enhanced root development facilitates deeper penetration into the soil for nutrient absorption, ultimately improving nitrogen assimilation within the plant (Xia et al., 2024). Correlation analysis revealed a highly significant negative correlation between bulk density and root vitality (**Figure 4**). Nitrogen fertilizers must undergo hydrolysis by urease to convert into ammonium nitrogen, which plants can absorb. Our results show that all experimental treatments significantly increased urease activity during the root elongation stage compared to CK treatment (**Figure 2A**), consistent with the findings of Li et al. (2023), who reported that both lime and biochar enhance urease activity in acidic soils. However, during the vigorous growth and topping stages, no significant changes in urease activity were observed, likely due to stabilization in the hydrolysis process of fertilizers as tobacco growth progressed, with other nitrogen cycle processes remaining active.

Nitrification, a primary cause of soil acidification (Lu et al., 2014), is itself inhibited by soil acidity (Ye et al., 2022). For tobacco, nitrate nitrogen serves as the primary nitrogen source, and the nitrification process significantly impacts nitrogen utilization efficiency (He et al., 2017). In this study, compared to the B and L+B treatments, the L treatment exhibited a more pronounced nitrification potential in the rhizosphere soil during the vigorous growth stage, with the nitrification potential in the L+B treatment significantly higher than that in the B treatment (**Figure 2B**). This result aligns with the findings of Teutscherova et al. (2018; 2023), who suggested that lime application can enhance soil nitrification rates by promoting the proliferation of nitrifying bacteria. Conversely, some studies argue that biochar’s adsorption of ammonium nitrogen may reduce nitrification rates (Yang et al., 2015). Thus, biochar primarily enhances nitrogen absorption and assimilation in plants by improving the soil environment, whereas lime stimulates nitrification, increasing the supply of available nitrogen. The combined application of both amendments effectively retains the advantages of each.

These findings prompted further investigation into nitrogen content and the microbial communities involved in rhizosphere nitrogen cycling in tobacco fields. Soil acidification typically leads to nutrient immobilization, reducing nutrient availability and thereby limiting plant-accessible nutrients (Adekiya et al., 2024). In this study, all experimental treatments significantly increased alkali-hydrolyzable nitrogen and nitrate nitrogen content compared to the CK treatment (**Figures 3A, C**), indicating that amendments effectively mitigate nutrient immobilization in soil. Additionally, the B and L+B treatments significantly increased microbial biomass nitrogen compared to the L treatment (**Figure 3D**). Cheng et al. (2017) found that enhancing microbial assimilation of inorganic nitrogen and converting it into microbial biomass nitrogen is an essential mechanism for reducing soil inorganic nitrogen loss. This finding aligns with Wang and Lu (2024), who indicated that biochar improves the growth environment for microorganisms, thereby enhancing microbial nitrogen assimilation capacity (Hammer et al., 2014). Interestingly, although the L treatment exhibited a higher nitrification potential, its nitrate nitrogen content was significantly lower than that in the B and L+B treatments. This may be due to the tendency of nitrate nitrogen to leach, whereas biochar can adsorb nitrate ions onto its anionic exchange sites, thus reducing denitrification efficiency and minimizing nitrate loss (Mandal et al., 2016). Correlation analysis revealed a significant positive relationship between soil pH, exchangeable cation concentrations, and both nitrogen utilization efficiency and nitrogen assimilation capacity in plants. These factors also showed a strong positive correlation with available nitrogen content in the soil. This suggests that increases in soil pH and exchangeable cation concentrations are key physicochemical factors promoting plant-soil nitrogen transformation. However, no significant correlation was observed between nitrification potential and nitrogen utilization efficiency, nor between nitrification potential and nitrate nitrogen content (**Figure 4**), implying that the mechanisms by which lime and biochar enhance nitrogen utilization efficiency in acidic soils may be more complex than previously understood.

The results indicate that lime is particularly effective in promoting nitrification, while biochar excels in enhancing microbial nitrogen assimilation. To further explore changes in rhizosphere nitrogen-cycling microbial communities and their functions, we performed high-throughput sequencing and used the PICRUSt2 tool to annotate the nitrogen cycling functions of bacterial communities based on the KEGG database. The results revealed that all experimental treatments significantly increased the functional abundance of nitrogen fixation compared to the CK treatment, with no significant difference observed between the L and B treatments (**Figure 5B**). This enhancement may be due to both lime and biochar supplying sufficient cations to meet the trace element requirements of nitrogen-fixing microorganisms (Nishio and Okano, 1991; Bossolani et al., 2020). Consistent with these findings, both the L and L+B treatments significantly increased the functional abundance of nitrification compared to the B and CK treatments. Additionally, the B and L+B treatments significantly enhanced the functional abundance of the assimilatory nitrate reduction process compared to the L and CK treatments. Both the B and L+B treatments also significantly increased the functional abundance of denitrification compared to the L and CK treatments, with the B treatment showing significantly higher levels than the L+B treatment (**Figure 5B**). This may be explained by the higher nitrate nitrogen content in the soil, which provides abundant substrates for denitrifying microorganisms (Chen et al., 2015).

Furthermore, the B and L+B treatments significantly increased the functional abundance of dissimilatory nitrate reduction compared to the L and CK treatments (**Figure 5**). Wan et al. (2023) highlighted that dissimilatory nitrate reduction is a key mechanism for retaining available nitrogen in soils. This process converts nitrate nitrogen, which is prone to being transformed into nitrous oxide (N_2_O), into ammonium nitrogen, reintegrating it into the nitrification cycle. Moreover, dissimilatory nitrate reduction and denitrification processes are closely coupled (Stein and Klotz, 2016), providing a reasonable explanation for the observed increase in denitrification functionality. Pearson correlation analysis revealed a significant positive correlation between tobacco nitrogen utilization efficiency and nitrogen fixation, as well as a significant positive correlation with dissimilatory nitrate reduction. Additionally, dissimilatory nitrate reduction showed a significant positive correlation with alkali-hydrolyzable nitrogen and nitrate nitrogen content in the soil (**Figure 6**). These results suggest that lime application primarily activates nitrification to enhance nitrate nitrogen production, while biochar enhances dissimilatory nitrate reduction functionality to retain more nitrogen. Therefore, the combined use of lime and biochar integrates the benefits of both amendments, thereby improving overall nitrogen cycling in the soil.

## 5 Conclusion

The findings of this study indicate that the application of either lime or biochar can alleviate soil acidification and increase the concentration of exchangeable cations, thereby improving nitrogen utilization efficiency in tobacco plants. Notably, biochar (B) demonstrated significantly more pronounced effects compared to lime (L). An integrated analysis of soil available nitrogen content and rhizosphere microbial community functions revealed that while lime was more effective in promoting nitrification within the soil nitrogen cycle, biochar treatment enhanced dissimilatory nitrate reduction. This process promotes the formation of a “microcycle” between ammonium and nitrate nitrogen in the soil, thereby more effectively retaining available nitrogen. This mechanism is key to understanding why biochar outperforms lime in enhancing crop nitrogen utilization. Moreover, the combined application of lime and biochar (L+B) synergistically integrates the benefits of both amendments, resulting in outcomes comparable to those achieved with either amendment alone (L or B). These results provide a significant theoretical foundation for the combined use of lime and biochar as an effective strategy to mitigate soil acidification. Future research should focus on exploring the synergistic effects of these amendments under different soil types and climatic conditions, as well as determining the optimal application ratios to establish more scientifically robust soil management strategies.

## Data Availability

The original contributions presented in the study are included in the article/**Supplementary Material**. Further inquiries can be directed to the corresponding authors.
