# Supplementary material for: Comparative and synergistic impacts of lime and biochar on soil properties, nitrogen transformation, and microbial function in acidic soils under tobacco cropping
Source: Front Plant Sci. 2025 Feb 7;16:1530128. doi: 10.3389/fpls.2025.1530128 (PMC11842323; doi:10.3389/fpls.2025.1530128)
Supplement: Supplementary file 1 [file DataSheet1.docx]

| 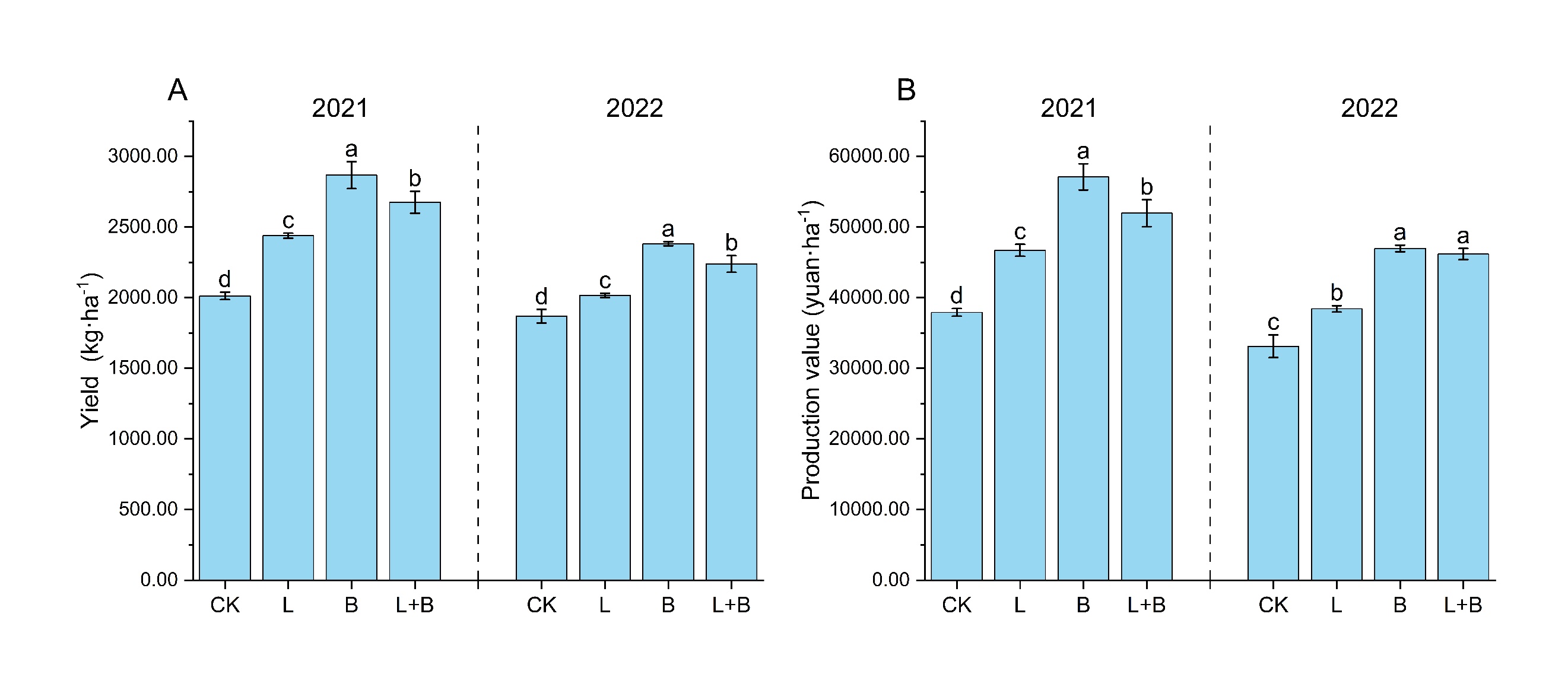 |
| --- |
| Fig.S1 Changes in tobacco yield (A) and production value (B). CK: control without soil amendment; L: lime-alone treatment; B: biochar-alone treatment; L+B: lime and biochar combined treatment. Data depicts means ± SD of three biological replicates. Significant differences between treatments (*P* < 0.05) are illustrated by different lowercase letters |

| 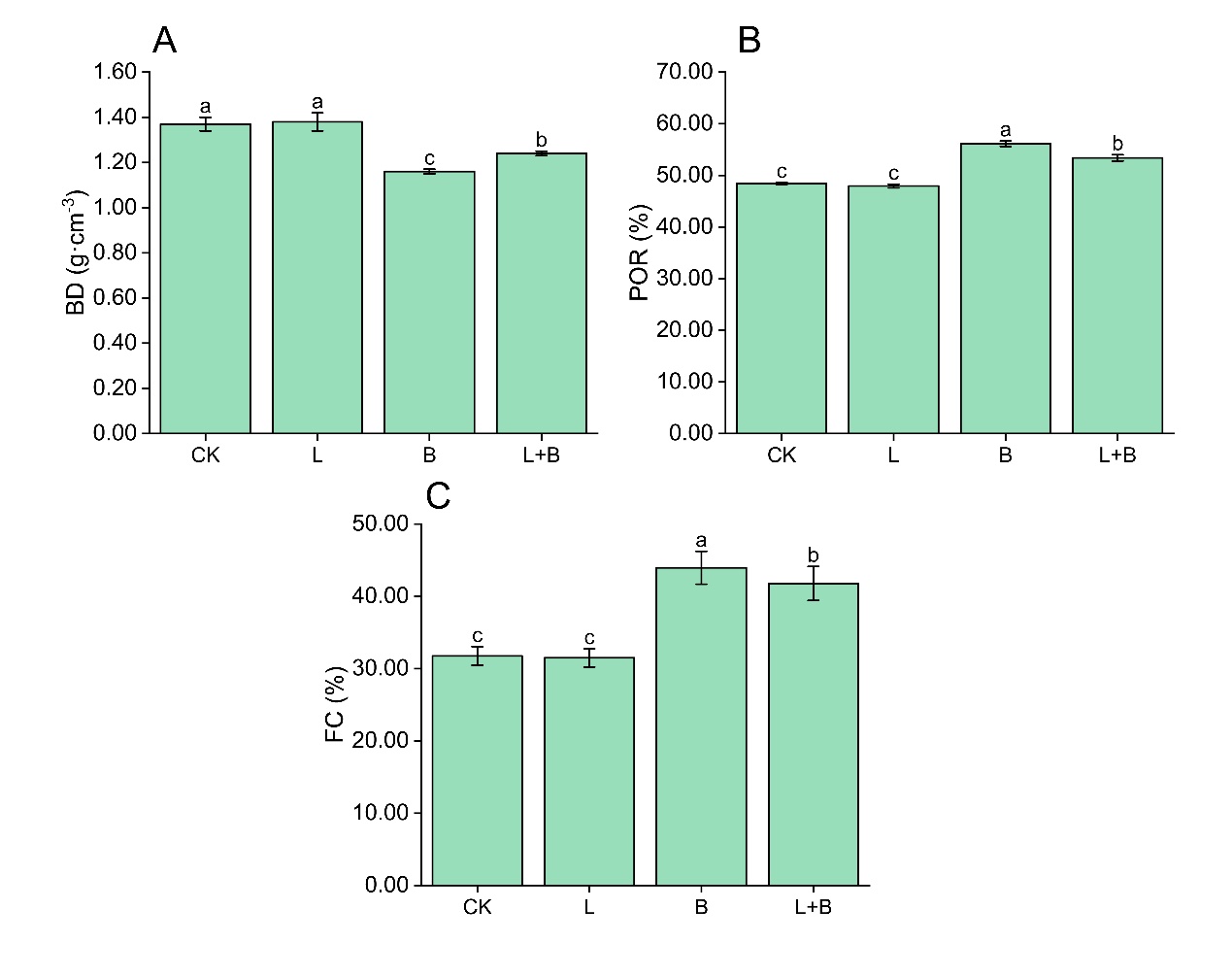 |
| --- |
| Fig. S2 Changes in soil bulk density (A), porosity (B), and field moisture capacity (C). BD: Bulk density; POR: Porosity; FC: Field moisture capacity; CK: control without soil amendment; L: lime-alone treatment; B: biochar-alone treatment; L+B: lime and biochar combined treatment. Data depicts means ± SD of three biological replicates. Significant differences between treatments (*P* < 0.05) are illustrated by different lowercase letters |

| Table S1 Changes in species diversity of tobacco rhizosphere bacterial communities under different soil treatments | | | | |
| --- | --- | --- | --- | --- |
|  | Observed OTUs | Ace | Chao1 | Shannon |
| CK | 4019.27±117.60b | 5601.81±90.12b | 5660.69±125.02b | 9.01±0.32b |
| L | 5070.67±179.72a | 6781.00±300.03a | 6766.23±268.57a | 9.95±0.18a |
| B | 4321.00±446.76b | 5874.61±460.03b | 5868.84±472.21b | 9.35±0.32b |
| L+B | 4875.33±293.01a | 6451.03±165.65a | 6482.99±329.78a | 9.90±0.29a |
| CK: control without soil amendment; L: lime-alone treatment; B: biochar-alone treatment; L+B: lime and biochar combined treatment. Data depicts means ± SD of three biological replicates. Significant differences between treatments (*P* < 0.05) are illustrated by different lowercase letters | | | | |

| 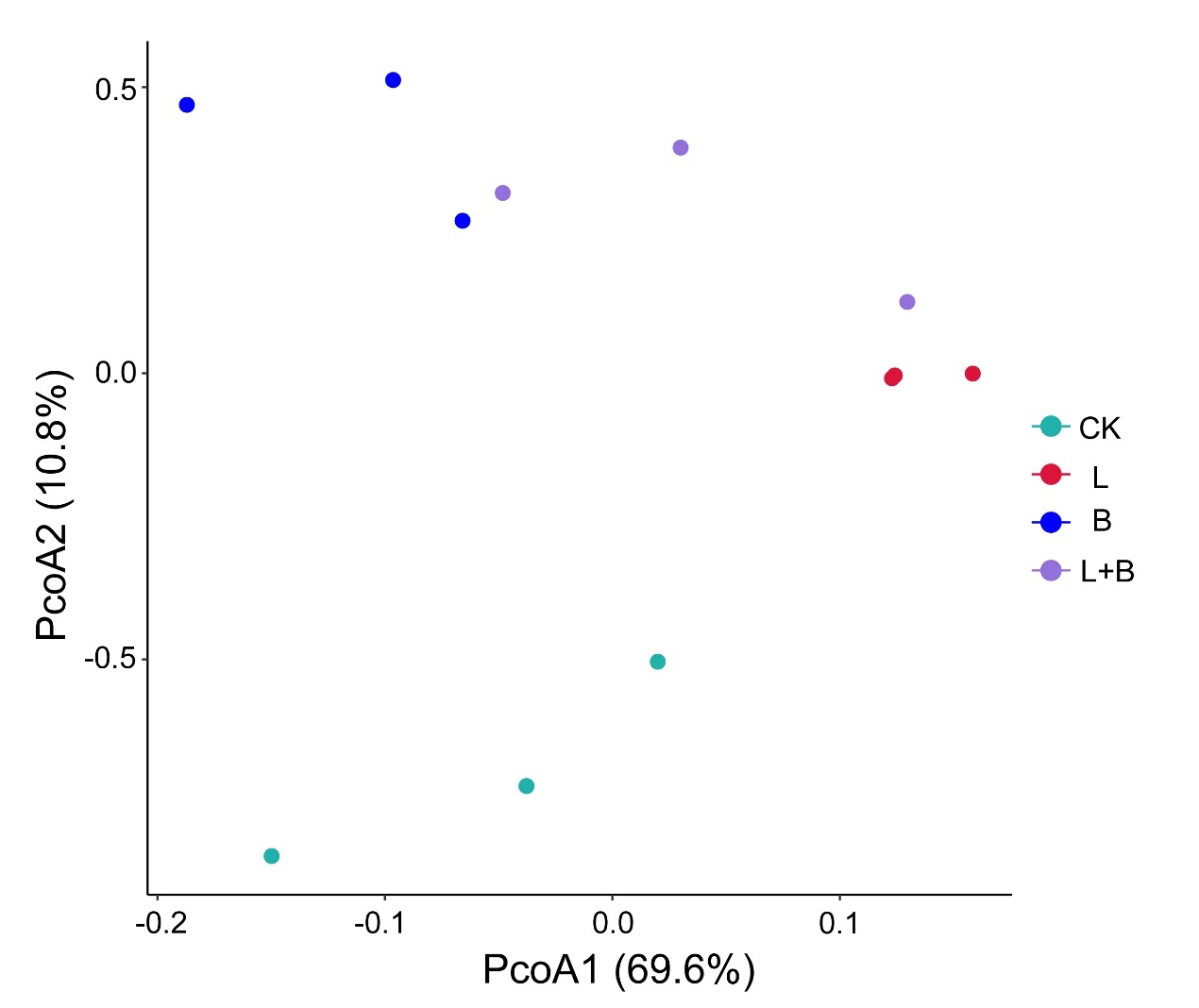 |
| --- |
| Fig.S3 Community species composition PCoA analysis based on Weighted-Unifrac distance. CK: control without soil amendment; L: lime-alone treatment; B: biochar-alone treatment; L+B: lime and biochar combined treatment. |

| 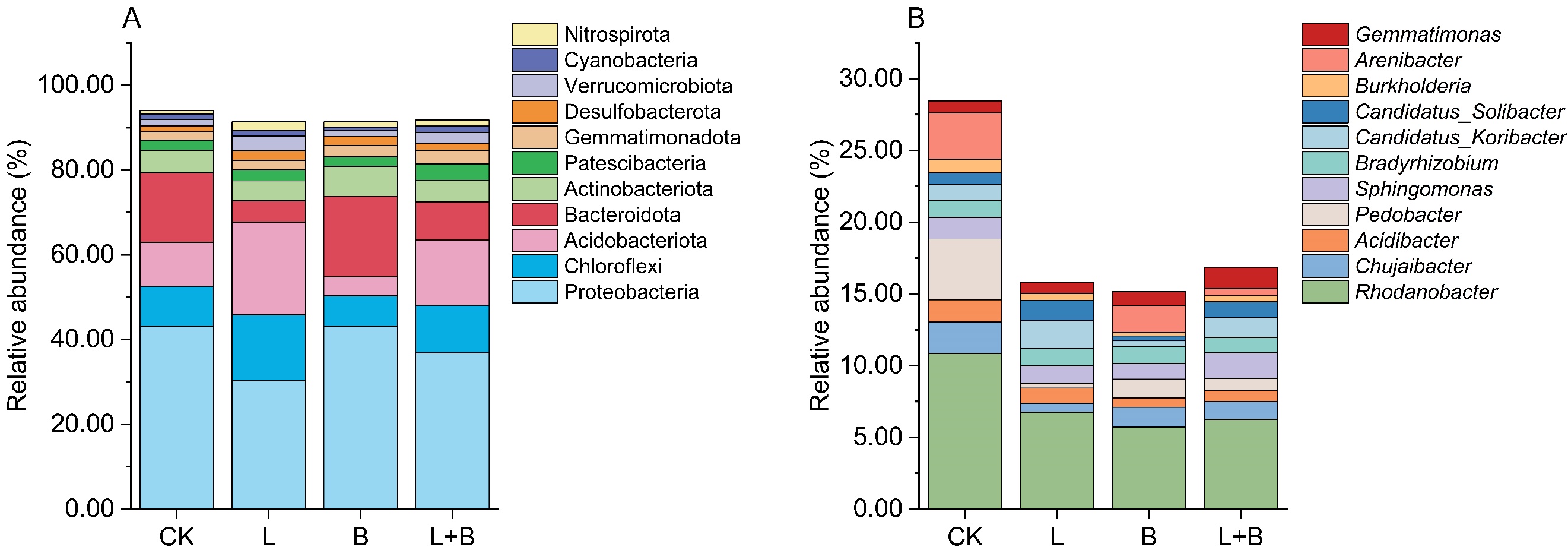 |
| --- |
| Fig.S4 Relative abundance of dominant phyla (A) and genera (B) in the tobacco rhizosphere under different treatments. CK: control without soil amendment; L: lime-alone treatment; B: biochar-alone treatment; L+B: lime and biochar combined treatment. |
